# Anxiety among children and adolescents during the COVID-19 pandemic in Europe: a systematic review protocol

**DOI:** 10.1186/s13643-023-02225-1

**Published:** 2023-04-11

**Authors:** Helena Ludwig-Walz, Indra Dannheim, Lisa M. Pfadenhauer, Jörg M. Fegert, Martin Bujard

**Affiliations:** 1 Federal Institute for Population Research (BiB), Wiesbaden, Germany; 2 Regional Innovative Centre of Health and Quality of Live Fulda (RIGL), Department of Nutrition, Food and Consumer Sciences, Fulda University of Applied Sciences, Fulda, Germany; 3 Institute for Medical Information Processing, Biometry and Epidemiology-IBE, Chair of Public Health and Health Services Research, LMU Munich, Munich, Germany; 4 Pettenkofer School of Public Health, Munich, Germany; 5 Department for Child and Adolescent Psychiatry and Psychotherapy, University Medical Center, Competence Domain Mental Health Prevention, Ulm, Germany; 6 Institute for Medical Psychology, Medical Faculty, University Heidelberg, Heidelberg, Germany

**Keywords:** Anxiety, COVID-19, Child, Adolescents, Europe, Systematic review

## Abstract

**Background:**

A growing number of studies point to a high mental health burden among children and adolescents during the COVID-19 pandemic, particularly concerning anxiety. However, the study quality and effect direction are heterogeneous in the existing primary studies with a lacking overview for the European continent. Therefore, this systematic review aims to critically synthesise the evidence regarding the impact of the COVID-19 pandemic on anxiety among children and adolescents in Europe compared to a pre-pandemic baseline.

**Methods:**

A systematic literature search will be performed in six databases (MEDLINE, EMBASE, PsycINFO, Cochrane Central Register of Controlled Trials, Web of Science, and WHO COVID-19 database) with a peer reviewed search strategy according to the evidence-based checklist Peer Review of Electronic Search Strategies (PRESS). Inclusion criteria are children and adolescents ≤ 19 years living in Europe and data report during the COVID-19 pandemic with an appropriate pre-pandemic baseline. Primary outcomes are general anxiety symptoms and clinically relevant anxiety rates. Risk of bias will be assessed using the ‘Risk of Bias in Non-randomised Studies of Exposure’ (ROBINS-E). Data extraction will systematically include information on study design, population characteristics, COVID-19 determinants, pre-pandemic baseline, diagnostic instruments and outcome. The certainty of evidence for each outcome will be evaluated by using the Grading of Recommendations Assessment, Development and Evaluation (GRADE) approach adapted to the use of non-randomised studies. All process steps will be performed independently by two reviewers; any discrepancies will be discussed and, if necessary, resolved by a third author. Also, subgroup analysis, sensitivity analysis, publication bias analysis, and meta-regression analysis, if applicable, will be performed. The systematic review was registered in the Prospective Register of Systematic Reviews (PROSPERO) and the protocol was prepared in accordance to the Preferred Reporting Items for Systematic review and Meta-Analysis Protocols (PRISMA-P) statement.

**Discussion:**

This systematic review will address the lack of a critical and comprehensive summary of findings on the COVID-19 pandemic impact on anxiety among children and adolescents in Europe. In addition, it aims to identify pandemic-policy differences, such as the effect of school-closures, and identify particularly vulnerable risk groups.

**Systematic review registration:**

CRD42022303714 (PROSPERO).

**Supplementary Information:**

The online version contains supplementary material available at 10.1186/s13643-023-02225-1.

## Background

The COVID-19 pandemic affects many public health (PH) fields. Besides disease rates, persistent symptoms (Long-COVID) and death, impacts on mental health aspects are essential with regard to short-term and long-term well-being [1, 2]. To keep incidence rates as low as possible, governments used various combinations of social isolation strategies [3–6]. However, compared to adults, children and adolescents (CA) represent a particularly vulnerable group and tend to be affected differently by the pandemic and social distancing policies such as school closures. On the one hand, the short-term health effects of COVID-19 infections on CA without comorbidities seemed to generally be mild, e.g. clinically mild disease or asymptomatically infection [7–9]. On the other hand, however, a growing number of studies point to a high mental health burden among the youth during the pandemic; particularly regarding anxiety [7, 10–12] as the most prevalent mental health disorder among young people in Europe and the leading cause of years lived with disability among mental health conditions [13, 14].

In studies of earlier pandemics and disease-related quarantine, associations between loneliness and isolation with mental health problems such as anxiety are already well described in CA [15, 16]. Many of the exposed children began using mental health services [17, 18]. Hence, CA seemed to be particularly vulnerable to isolation or loneliness which could lead to an increase in mental health impacts through COVID-19 containment measures [11, 19]. According to UNICEF pre-pandemic estimates, the prevalence of mental disorders for boys and girls aged 10–19 in Europe was 16.3% in 2019 [16]. Further, the World Health Organization (WHO) described anxiety disorders as the most prevalent in this age group with profound consequences on physical and mental health in later life [20].

Within the ongoing COVID-19 pandemic, the number of primary studies regarding the effects of the pandemic on anxiety among CA is rapidly increasing. However, the existing studies provide partially contradictory findings [21, 22], have different results regarding the magnitude of anxiety and used heterogeneous diagnostic instruments [10, 12]. Up to now existing systematic reviews primarily focus on the general population [23] or the global prevalence of anxiety among CA [8, 10]. Since the COVID-19 pandemic confronted populations in Europe with several waves and national state governments reacted with heterogenous contact restrictions like lockdowns, school/kindergarten closures, quarantine orders, decreased peer interactions etc., a summary within a European context could allow a differential view on a potential increase of anxiety symptoms during the course of the pandemic. A recent published meta-analysis of our research group regarding the changes of depression symptoms in CA in Europe during the COVID-19 pandemic highlights an overall increase as well as a dose–response relationship of restriction measures and general depression symptoms [24]. At present, no systematic review specifically addresses the changes during COVID-19 pandemic for anxiety among CA on the European continent. Therefore, the aim of this systematic review (SR) is to critically synthesise the evidence regarding the impact of the COVID-19 pandemic on anxiety symptoms among CA in Europe compared to a pre-pandemic baseline. To this end, the proposed systematic review will address the following research objectives:

1. Estimation the change of general anxiety symptoms and clinically relevant anxiety rates among CA in Europe before and during the COVID-19 pandemic in total and pre-defined subgroups (in particular regarding age and gender);
2. Evaluation the impact of COVID-19-related measures stringency on general anxiety symptoms and clinically relevant anxiety rates, using the validated Oxford COVID-19 Stringency index [6];
3. Identification of vulnerable groups among CA;
4. Outline the clinical relevance of the available results.

In this protocol of the planned SR, the used methods will be described.

## Methods

The SR was registered on the International Prospective Register of SR (PROSPERO; CRD42022303714) [25]. This protocol is prepared in accordance with the Preferred Reporting Items for Systematic review and Meta-Analysis Protocols (PRISMA-P) statement [26] (Appendix 1); the PROSPERO record will be updated regularly. Any deviations from the protocol will be noted in the final SR. The final SR will be conducted according to updated PRISMA statement [27] and will follow the guidelines of the actual Cochrane Handbook for SR [28] and the Joanna Briggs Institute (JBI) Manual for Evidence Synthesis [29], as far as possible.

### Eligibility criteria and information

Based on the examination of an environmental exposure, namely the COVID-19 pandemic, the research question was formulated within a Population-Exposure-Comparison-Outcome (PECO) scheme [30], see Table 1.

**Table 1:** Research question according to the Population-Exposure-Comparison-Outcome (PECO) scheme

Category	Definition
**P**opulation	Children and adolescents ≤ 19 years in Europe
**E**xposure	COVID-19 pandemic
**C**omparison	Pre-pandemic baseline
**O**utcome	Anxiety

The eligibility criteria, divided into inclusion and exclusion criteria, were conducted in accordance to the PECO scheme and are presented in Table 2 with further categories.

**Table 2:** Inclusion and exclusion criteria according to the Population-Exposure-Comparison-Outcome (PECO) scheme

Category	Inclusion criteria	Exclusion criteria
**P**opulation	Children and adolescents ≤ 19 years^a^ of any gender^b^ and any ethnicity in Europe Europe will be defined as European continent according to the definition of World Health Organization (WHO) Regional Office for Europe [31]	Studies with population samples with > 19 years or mixed population samples of children, adolescents and/or adults Samples of children with preexisting psychiatric diagnoses Countries that are not included in the WHO overview [31]
**E**xposure	Data collection within COVID-19 pandemic	Other previous pandemics Studies that analysed anxiety due to the use of alcohol or other drugs
**C**omparison	Pre-pandemic baseline	No comparison Comparisons of two time points within the COVID-19 pandemic
**O**utcome	Anxiety, based on self-reports or (validated) measurements	Other outcomes
Effect measures	All effect measures	–
Study design	Primary studies or reports	Reviews, systematic review, meta-analysis, case studies
Language	All languages	–
Time frame	Publication as of November 1, 2019^c^	Other time frames
Publication status	Published studies, grey literature, pre-prints, conference abstracts	Other publication status
Species	Human studies	Animals studies

^a^The cut-off of ≤ 19 years was chosen in accordance with the World Health Organization report [20]

^b^In accordance with the Sex and Gender Equity in Research (SAGER) guideline we assume the term ‘gender’ which refers to socially constructed roles, behaviours and identities of females, males, and gender-diverse people [32]

^c^According to the PubMed COVID-19 article filter [33]

### Search strategy

The search strategy includes the following databases: MEDLINE (PubMed), Embase, PsycINFO, Cochrane Central Register of Controlled Trials (CENTRAL), Web of Science and WHO COVID-19 database (also including pre-prints). Also, study registries (e.g. PROSPERO), relevant grey literature (e.g. government reports), related articles, congress submissions, websites of key organisations, reference lists of included articles and previous published reviews will be screened.

Translating the research question into a search string was performed in accordance with the guideline for Peer Review of Electronic Search Strategies (PRESS) [34]. Development of the database specified search strings occurred using validated or recommended search filters where possible (e.g. for identifying pediatric studies in PubMed [35], search strings for COVID-19 records in PubMed [33, 36], search filters offered by the InterTASC Information Specialists’ Subgroup Search Filter Resource [37]; in parts adapted). Both free-text and subject headings (e.g. Medical Subject Headings [MeSH], Emtree) will be used in combination with the adequate use of the Boolean operators ‘AND’ and ‘OR’. The search strategy was peer reviewed by an expert in conducting SR in health sciences according to the evidence-based checklist PRESS Evidence-Based Checklist [34] before the searches will be run to ensure a high-quality search strategy (search submission and peer review assessment are attached in Appendix 2 and 3). The draft search strategy for PubMed is presented in Appendix 4.

### Study records

Study selection, in accordance to the inclusion and exclusion criteria in Table 2, will be conducted in three steps: (1) duplicates removal; (2) screening at title and abstract level; and (3) screening the full text. Duplicate removal will be conducted with assistance of the recommended *EPPI-Reviewer Web software* [38]. Two independent reviewers (HLW, ID) will screen the studies in step (2) and (3); any discrepancies will be discussed and, if necessary, resolved by a third author (MB). Several publications with an equal or similar study population and equal measurement points during the pandemic will be considered once; studies with, e.g. smaller sample sizes will be excluded. Studies of the same study population with various pandemic measurement points will be considered individually. The reasons for study exclusion in step 3 will be reported in the Appendix of the final study. All screening procedures will be presented using the PRISMA flow diagram [27]. Data management will be organised by the software *Citavi 6*.

### Data collection

Data extraction will be conducted by two review authors (HLW, ID) using specially developed tabular data collection forms (‘Characteristics of included studies’ and ‘Summary of effect estimates’ tables are planned) [28]. These forms will be pilot tested with about one third of the included studies by both authors transferring the data independently from the studies and discussing possible discrepancies. Remaining data extraction will be completed by one reviewer (HLW) and verified by the other (ID). Any discrepancies between the two reviewers will be discussed extensively and, if necessary, resolved by a third author (MB). Study authors will be contacted in case of uncertainties regarding the published data. Further, several authors will be contacted to provide additional unpublished study data to expand the data basis and, if possible, to be able to perform (more detailed) subgroup analyses (e.g. gender- or age-stratified data).

For each included study the information of five categories (study information, population and setting, COVID-19 determinants, pre-pandemic baseline, outcomes) will be extracted for an overview of the study characteristics in a ‘Characteristics of included studies’ table, as shown in Table 3.

**Table 3:** Planned data for extraction

Category	Planned data for extraction
Study information	- First author
	- Year of publication
	- Country
	- Study type
Population and setting	- Sample size, % female
	- Age (mean, median or range)
COVID-19 determinants	- Time point (month/year) of data measurement
	- Policy restrictions in the measurement period, described by using the Oxford COVID-19 Stringency Index and the School Closure Index [6] as a proxy indicator
Pre-pandemic baseline	- Time point (month/year) of data measurement
	- Link between pre-pandemic and during pandemic population (same population, similar population, cross-sectional population sample)
Outcomes	Definition of outcome (e.g. means, SD, events)
	- Diagnostic instrument
	- Psychometric properties of the diagnostic instrument
	- Symptom reporter (self-reported, parent-report or physician-reported)

The primary outcomes are

- General anxiety symptoms
- Clinically relevant anxiety rates

General symptoms are defined as the general measurement of anxiety symptoms (mostly continuous measurements). Clinically relevant rates are defined as measurements with a clinical cut-off or in a medical setting (ICD-reports). Changes will be calculated as differences between scores with standard deviation (general symptoms) or ratios (clinically relevant anxiety symptoms; see “Data synthesis” section for further information). We assume that definitions and diagnostic instruments will vary across studies, so a wide range of definitions, measurement instruments and symptom reporter will be accepted. No restrictions will be set on the number of measurements during the COVID-19 pandemic. If data of pre-pandemic measurements will be available at multiple time points, only data of the latest possible time point will be used for effect estimate calculation. If present, both unadjusted and adjusted effects estimates will be extracted, whereby adjusted values will be preferred in the case of pooling (see “Data synthesis” section). The effect estimate will be provided with a 95% confidence interval (CI). No second outcomes will be considered.

We will put a special focus on the impact of pandemic-related restrictions on anxiety at CA (research objective 2) by using the validated Oxford COVID-19 Stringency Index [6]. The index is calculated as a score from nine categories: school closures, workplace closures, cancellation of public events, restrictions on public gatherings, closures of public transport, stay-at-home requirements, public information campaigns, restrictions on internal movements and international travel controls; it ranges from 0 (no restrictions) to 100 (most stringent restrictions). We will calculate for each study measurement period a mean score. Further, we will define three cut-off points in accordance with the COVIDSurg Collaborative [39]: light restrictions (index < 20), moderate lockdowns (index 20–60) and full lockdowns (index > 60). In addition, we plan to consider specifically the School Closure Index (also included in the Oxford COVID-19 Stringency Index) which records closings of schools. The range of the School Closure Index comprises 0 to 3: 0 for no measures, 1 for recommended closings or changes in school operations, 2 for partially school closures and 3 for closing of all school levels [6, 40]. Therefore, we will define the following cut-offs points: no or few alterations compared to a pre-COVID-19 situation (index < 2) and partial or full school closure (index ≥ 2) [24].

### Risk of bias assessment

Based on preliminary searches and previously published systematic reviews [8, 10, 24], we expected mainly observational studies. Therefore, two review authors (HLW, LMP) will independently assess the risk of bias applying the current launched instrument ‘Risk Of Bias In Non-randomised Studies of Exposure’ (ROBINS-E) [41]. The ROBINS-E development followed the standards of the ‘Risk Of Bias In Non-Randomised Studies of Interventions’ (ROBINS-I) tool, in which RoB assessments are made within a set of ‘signalling questions’ within seven bias domains, including (1) risk of bias due to confounding, (2) risk of bias arising from measurement of the exposure, (3) risk of bias in selection of participants into the study, 4) risk of bias due to post-exposure interventions, 5) risk of bias due to missing data, 6) risk of bias arising from measurement of the outcome, and 7) risk of bias in selection of the reported result [42]. Judgments for each RoB item could be ‘low’, ‘some concerns’, ‘high RoB’, or ‘very high RoB’. Also, an overall judgment regarding the total RoB quality will occur. For further analysis we aim to differentiate between studies classified as having low/some concerns (= low) RoB and high RoB/very high RoB (= high) RoB (see “Data synthesis” section for further information). RoB assessments will be visualised as ‘traffic light’ plots of the domain-level judgements for each individual result and ‘weighted bar’ plots of the distribution of risk-of-bias judgements within each bias domain, using the tool *robvis* [43].

### Data synthesis

The ‘Summary effect estimates’ tables will be presented and described for each study and outcome estimate, grouped by country and RoB (see ‘Data collection’ section). We will conduct the decision to combine (or not) the results of the individual studies (meta-analysis) in accordance with the assessment of clinical and methodological heterogeneity by considering gender, age, pandemic-related restrictions and RoB [44]. Where data will be pooled using meta-analysis, we will assess the degree of statistical heterogeneity by visual inspection of forest plots and applying chi^2^ test and *I*^2^ statistic. A low p value within chi^2^ test (or a large chi^2^ statistic relative to its degree of freedom) will be considered as an indication of heterogeneity but will be interpreted with caution when only few studies can be included or the studies have small sample sizes [45]. We will consider an I^2^ value of 50% or more to represent substantial levels of heterogeneity, but will interpret this value in light of the size and direction of effects and the strength of the evidence for heterogeneity. Where heterogeneity will be found in pooled effect estimates an explanation of the source of heterogeneity will be pursued by subgroup analyses, sensitivity analyses and/or meta-regression analyses [45]. To conduct a meta-regression analysis a minimum of 10 studies should be available per examined covariate [45].

Based on preliminary analysis, it is anticipated that general anxiety symptoms will be reported as continuous outcomes. If studies will use different outcome measures to assess general anxiety symptoms, the standardised mean difference (SMD) with a 95% CI will be used as a summary statistic (recommend by the Cochrane Handbook [46]). It can be further assumed that clinically relevant anxiety rates will be reported as dichotomous outcomes (odds ratio or risk ratio). The meta-analyses of both the continuous and dichotomous data will be performed based on the random-effect model (due to anticipated between-study heterogeneity) using the inverse-variance method with the ‘DerSimonian and Laird’ approach (to minimise the imprecision of the pooled effect estimate). If standard deviations are missing, we will calculate them from *p* values, CIs or standard errors, if available or contact the study authors [28]. If data for general anxiety symptoms will be reported as dichotomous data, it is planned to homogenise these data [47]. For the expression of dichotomous data as SMD the recommended formula by Chinn [45, 48] will be used. Results from adjusted analysis will get preference in the meta-analyses to provide more careful estimates. When both parent and self-rated data will be provided the self-rated data will be selected for meta-analysis [49]. Results of the meta-analysis will be illustrated using forest plots.

Study data will be categorised (e.g. in an excel-matrix) to decide which data sets are similar enough for a quantitative pooling, which subgroups are feasible and which data types (continuous or dichotomous data) are available (considering clinical and methodological heterogeneity). RoB will be given special attention by performing separate calculations, if possible, for low and high RoB studies as well as by calculating the overall summarising effect. The following subgroup analyses are planned, assuming that sufficient data from low RoB studies are available:

- Outcome: general anxiety symptoms and clinically relevant anxiety rates;
- Demographic: gender and age;
- Contextual: full versus moderate lockdown (Oxford Stringency Index > 60 versus ≤ 60), school closures versus no school closures (School Closure Index ≥ 2 versus < 2), social status (high versus low social status) and education (high versus low educational level).

For research objective number no. 4 a medical interpretation of the change of clinically relevant anxiety rates will occur considering further relevant clinical aspects (ensured by JMF and MB).

The analyses will be conducted with Review Manager 5.4.1 [50] and/or R Studio 4.2.1 [51].

If a statistical pooling (meta-analysis) appears to be inappropriate, e.g. if study designs differ considerably, a tabular, graphical or narrative synthesis will be provided [52].

### Sensitivity analysis

To determine whether the pooled results are robust, sensitive analyses will be conducted. This includes the repetition of the meta-analysis with different comparison categories [28, 53], planned are comparisons between low and high RoB studies, different study types, converted/unconverted and adjusted/unadjusted effect estimates (removing those studies with converted/unadjusted effect estimates).

### Publication bias

The systematic review will also address RoB due to missing results in a synthesis. Graphical and statistical methods will be used to provide information about the extent of missing results. Funnel plots will be generated and visually interpreted for signs of asymmetry, which could indicate that publication bias is present [54, 55]. When at least 10 studies of different sample size will be included in a meta-analysis, the Eggers’ test will be used to test for funnel plot asymmetry[56].

### Certainty of evidence

The certainty of evidence for each outcome will be evaluated by using the ‘Grading of Recommendations Assessment, Development and Evaluation’ (GRADE) approach adapted to the use of non-randomised studies [57]. Five domains for downgrading the certainty of evidence are considered in GRADE: RoB, inconsistency, indirectness, imprecision, and publication bias. Also, an upgrading is possible through consideration of three further domains: large effects, dose response, and opposing plausible residual bias and confounding [58]. The use of the RoB instrument for non-randomised studies will allow to start at ‘high’ initial certainty of evidence within GRADE [53]. For all domains, we will follow a transparent approach of applying detailed criteria for downgrading or upgrading. GRADE finally specifies four levels of the certainty for a body of evidence for each outcome: high (further research is very unlikely to change our confidence in the estimate of effect), moderate (further research is likely to have an important impact on our confidence in the estimate of effect and may change the estimate), low (further research is very likely to have an important impact on our confidence in the estimate of effect and is likely to change the estimate), or very low (very uncertain about the estimate of effect). The certainty of evidence will be report for each outcome in a ‘Summary of findings’ table, supported by evidence profiles with more detailed explanations [58].

## Discussion

This protocol aims to provide a description of the research design and used methods of the SR addressing the real impact of the COVID-19 pandemic on anxiety among CA in Europe in contrast to many clinical and epidemiological observations without pre-pandemic baseline. The results of the SR will provide relevant evidence in order to address the gap in the literature with a high-quality methodological approach.

As a strength of the systematic review it can be pointed out that only methods and instruments that have already been tested and approved will be used. Although the study design will be not limited, it can be assumed that in particular observational studies will be included in the final systematic review; this might restrict the certainty of evidence. At present, there is no applied guideline for the preparation of a systematic review for observational studies or other study designs besides RCTs; however, some guidelines are in preparation [59–61]. The Cochrane Handbook [28] is often cited as the ‘gold standard’ for preparing SR. It contains important information on the preparation of clinically relevant search strategies (e.g. PRESS review), the synthesis of the results and the subsequent assessment of the RoB and the certainty of evidence (GRADE), but offers only few descriptions of how to prepare and conduct a SR with observational studies. In addition, the JBI provides comprehensive guides for conducting a variety of review types, including SR, scoping reviews, and umbrella reviews to address health-related questions [62]. 

Potential limitations of this SR could be the heterogeneity of studies, methodological approaches, and probable reduced number of studies due to urgency of a pre-pandemic baseline (see Table 2). Nonetheless, given a disparate and partly contradictory state of research, based on heterogeneous diagnostic instruments, age groups, pandemic situation, and country, the SR will provide a systematic assessment of the impact of the COVID-19 pandemic and its social distancing policies on anxiety among CA in Europe.

## Supplementary Information

**Additional file 1: Appendix 1.** Preferred Reporting Items for Systematic Review and Meta-analysis (PRISMA-P) 2015 checklist. **Appendix 2.** Peer Review of Electronic Search Strategies (PRESS) search submission form. **Appendix 3.** Peer Review of Electronic Search Strategies (PRESS) peer review assessment form. **Appendix 4.** Search strategy draft for PubMed. 

## Data Availability

Not applicable.

## Abbreviations

CA: Children and adolescents
COVID-19: Coronavirus disease 2019
GRADE: Grading of Recommendations Assessment, Development and Evaluation
PECO: Population-Exposure-Comparison-Outcome
PH: Public health
PRESS: Peer Review of Electronic Search Strategies
PRISMA-P: Preferred Reporting Items for Systematic review and Meta-Analysis Protocols
PROSPERO: Prospective Register of Systematic Reviews
RoB: Risk of bias
SR: Systematic review

## References

[1] World Health Organization. Improving early childhood development: WHO Guideline. 2020. https://www.who.int/publications/i/item/97892400020986. Accessed 29 Aug 2022. 32200595

[2] World Health Organization (2021). Comprehensive Mental Health Action Plan 2013–2030.

[3] Gianino MM, Nurchis MC, Politano G, Rousset S, Damiani G (2021). Evaluation of the strategies to control COVID-19 pandemic in four European countries. Front Public Health.

[4] Brauner JM, Mindermann S, Sharma M, Johnston D, Salvatier J, Gavenčiak T (2021). Inferring the effectiveness of government interventions against COVID-19. Science.

[5] Woskie LR, Hennessy J, Espinosa V, Tsai TC, Vispute S, Jacobson BH (2021). Early social distancing policies in Europe, changes in mobility & COVID-19 case trajectories: Insights from Spring 2020. PLoS One.

[6] Hale T, Angrist N, Goldszmidt R, Kira B, Petherick A, Phillips T (2021). A global panel database of pandemic policies (Oxford COVID-19 Government Response Tracker). Nat Hum Behav.

[7] Gaythorpe KAM, Bhatia S, Mangal T, Unwin HJT, Imai N, Cuomo-Dannenburg G (2021). Children's role in the COVID-19 pandemic: a systematic review of early surveillance data on susceptibility, severity, and transmissibility. Sci Rep.

[8] Viner RM, Mytton OT, Bonell C, Melendez-Torres GJ, Ward J, Hudson L (2021). Susceptibility to SARS-CoV-2 infection among children and adolescents compared with adults: a systematic review and meta-analysis. JAMA Pediatr.

[9] Tsankov BK, Allaire JM, Irvine MA, Lopez AA, Sauvé LJ, Vallance BA, Jacobson K (2021). Severe COVID-19 Infection and pediatric comorbidities: a systematic review and meta-analysis. Int J Infect Dis.

[10] Racine N, McArthur BA, Cooke JE, Eirich R, Zhu J, Madigan S (2021). Global Prevalence of Depressive and Anxiety Symptoms in Children and Adolescents During COVID-19: A Meta-analysis. JAMA Pediatr.

[11] Fegert JM, Vitiello B, Plener PL, Clemens V (2020). Challenges and burden of the Coronavirus 2019 (COVID-19) pandemic for child and adolescent mental health: a narrative review to highlight clinical and research needs in the acute phase and the long return to normality. Child Adolesc Psychiatry Ment Health.

[12] Viner R, Russell S, Saulle R, Croker H, Stansfield C, Packer J (2022). School closures during social lockdown and mental health, health behaviors, and well-being among children and adolescents during the first COVID-19 wave: a systematic review. JAMA Pediatr.

[13] Castelpietra G, Knudsen AKS, Agardh EE, Armocida B, Beghi M, Iburg KM (2022). The burden of mental disorders, substance use disorders and self-harm among young people in Europe, 1990–2019: Findings from the Global Burden of Disease Study 2019. Lancet Reg Health Eur.

[14] Bruckmayer M, Phillips W (2021). Children and mental health: preventive approaches to anxiety and depression: European platform for investing in children.

[15] Danneel S, Nelemans S, Spithoven A, Bastin M, Bijttebier P, Colpin H (2019). Internalizing problems in adolescence: linking loneliness, social anxiety symptoms, and depressive symptoms over time. J Abnorm Child Psychol.

[16] UNICEF. The state of the world's children: On my mind. Promoting, protecting and caring. Regional brief: Europe. 2021. https://www.unicef.org/eu/reports/state-worlds-children-2021. Accessed 29 Aug 2022.

[17] Sprang G, Silman M (2013). Posttraumatic stress disorder in parents and youth after health-related disasters. Disaster Med Public Health Prep.

[18] Vanhalst J, Klimstra TA, Luyckx K, Scholte RHJ, Engels RCME, Goossens L (2012). The interplay of loneliness and depressive symptoms across adolescence: exploring the role of personality traits. J Youth Adolesc.

[19] Loades ME, Chatburn E, Higson-Sweeney N, Reynolds S, Shafran R, Brigden A (2020). Rapid systematic review: the impact of social isolation and loneliness on the mental health of children and adolescents in the context of COVID-19. J Am Acad Child Adolesc Psychiatry.

[20] World Health Organization. Adolescent mental health. https://www.who.int/news-room/fact-sheets/detail/adolescent-mental-health. Accessed 29 Aug 2022.

[21] Luijten MAJ, van Muilekom MM, Teela L, Polderman TJC, Terwee CB, Zijlmans J (2021). The impact of lockdown during the COVID-19 pandemic on mental and social health of children and adolescents. Qual Life Res.

[22] Knowles G, Gayer-Anderson C, Turner A, Dorn L, Lam J, Davis S (2022). Covid-19, social restrictions, and mental distress among young people: a UK longitudinal, population-based study. J Child Psychol Psychiatry.

[23] PashazadehKan F, Raoofi S, Rafiei S, Khani S, Hosseinifard H, Tajik F (2021). A systematic review of the prevalence of anxiety among the general population during the COVID-19 pandemic. J Affect Disord.

[24] Ludwig-Walz H, Dannheim I, Pfadenhauer LM, Fegert JM, Bujard M (2022). Increase of depression among children and adolescents after the onset of the COVID-19 pandemic in Europe: a systematic review and meta-analysis. Child Adolesc Psychiatry Ment Health.

[25] Ludwig-Walz H, Dannheim I, Pfadenhauer LM, Fegert JM, Bujard M. Depression and anxiety among children and adolescents during the COVID-19 pandemic in Europe: a systematic review: PROSPERO 2022. 10.1186/s13643-023-02225-1 PMC10088269 37038242

[26] Moher D, Shamseer L, Clarke M, Ghersi D, Liberati A, Petticrew M (2015). Preferred reporting items for systematic review and meta-analysis protocols (PRISMA-P) 2015 statement. Syst Rev.

[27] Page MJ, McKenzie JE, Bossuyt PM, Boutron I, Hoffmann TC, Mulrow CD (2021). The PRISMA 2020 statement: an updated guideline for reporting systematic reviews. BMJ.

[28] Higgins JPT, Thomas J, Chandler J, Cumpston M, Li T, Page MJ, Welch VA, editor. Cochrane Handbook for Systematic Reviews of Interventions: version 6.3; 2022.

[29] Aromataris E, Munn Z, editors. JBI Manual for Evidence Synthesis: JBI; 2020.

[30] Morgan RL, Whaley P, Thayer KA, Schünemann HJ (2018). Identifying the PECO: A framework for formulating good questions to explore the association of environmental and other exposures with health outcomes. Environ Int.

[31] WHO Regional Office for Europe. Countries. 2022. https://www.euro.who.int/en/countries. Accessed 18 May 2022.

[32] Heidari S, Babor TF, de Castro P, Tort S, Curno M (2016). Sex and gender equity in research: rationale for the SAGER guidelines and recommended use. Res Integr Peer Rev.

[33] PubMed user guide. COVID-19 article filters: National Library of Medicine. https://pubmed.ncbi.nlm.nih.gov/help/#covid19-article-filters. Accessed 18 May 2022.

[34] McGowan J, Sampson M, Salzwedel DM, Cogo E, Foerster V, Lefebvre C (2016). PRESS Peer Review of Electronic Search Strategies: 2015 Guideline Statement. J Clin Epidemiol.

[35] Leclercq E, Leeflang MMG, van Dalen EC, Kremer LCM (2013). Validation of search filters for identifying pediatric studies in PubMed. J Pediatr.

[36] Lazarus JV, Palayew A, Rasmussen LN, Andersen TH, Nicholson J, Norgaard O (2020). Searching PubMed to retrieve publications on the COVID-19 pandemic: comparative analysis of search strings. J Med Internet Res.

[37] Glanville J, Lefebvre C, Wright K. ISSG search filter resource York (UK): The InterTASC Information Specialists' Sub-Group 2019. https://sites.google.com/a/york.ac.uk/issg-search-filters-resource/home. Accessed 18 May 2022.

[38] Thomas J, Graziosi S, Brunton J, Ghouze Z, O'Driscoll P, Bond M (2020). EPPI-Reviewer: advanced software for systematic reviews, maps and evidence synthesis.: EPPI-Centre Software.

[39] COVIDSurg Collaborative (2021). Effect of COVID-19 pandemic lockdowns on planned cancer surgery for 15 tumour types in 61 countries: an international, prospective, cohort study. Lancet Oncol.

[40] Phillips T (2022). Codebook for the Oxford Covid-19 Government Response Tracker: Codebook version 4.0.

[41] Higgins J, Morgan R, Rooney A, Taylor K, Thayer K, Silva R, Lemeris C, Akl A, Arroyave W, Bateson T, Berkman N, Demers P, Forastiere F, Glenn B, Hróbjartsson A, Kirrane E, LaKind J, Luben T, Lunn R, McAleenan A, McGuinness L, Meerpohl J, Mehta S, Nachman R, Obbagy J, O'Connor A, Radke E, Savović J, Schubauer-Berigan M, Schwingl P, Schunemann H, Shea B, Steenland K, Stewart T, Straif K, Tilling K, Verbeek V, Vermeulen R, Viswanathan M, Zahm S, Sterne J (ROBINS-E Development Group). Risk Of Bias In Non-randomized Studies - of Exposure (ROBINS-E): Launch version. 1 June 2022. https://www.riskofbias.info/welcome/robins-e-tool.

[42] Sterne JA, Hernán MA, Reeves BC, Savović J, Berkman ND, Viswanathan M (2016). ROBINS-I: a tool for assessing risk of bias in non-randomised studies of interventions. BMJ.

[43] McGuinness LA, Higgins JPT (2021). Risk-of-bias VISualization (robvis): An R package and Shiny web app for visualizing risk-of-bias assessments. Res Synth Methods.

[44] Ryan R, Cochrane Consumers and Communication Review Group (2016). Heterogeneity and subgroup analyses in Cochrane Consumers and Communication Group reviews: Planning the analysis at protocol stage.

[45] Deeks JJ, Higgins JP, Altman DG. Chapter 10: Analysing data and undertaking meta-analyses. In: Higgins JPT, Thomas J, Chandler J, Cumpston M, Li T, Page MJ, Welch VA, editor. Cochrane Handbook for Systematic Reviews of Interventions: version 6.3; 2022.

[46] Higgins JP, Li T, Deeks JJ. Chapter 6: Choosing effect measures and computing estimates of effect. In: Higgins JPT, Thomas J, Chandler J, Cumpston M, Li T, Page MJ, Welch VA, editor. Cochrane Handbook for Systematic Reviews of Interventions: version 6.3; 2022.

[47] Anzures-Cabrera J, Sarpatwari A, Higgins JP (2011). Expressing findings from meta-analyses of continuous outcomes in terms of risks. Statist Med.

[48] Chinn S (2000). A simple method for converting an odds ratio to effect size for use in meta-analysis. Statist Med.

[49] Ebesutani C, Bernstein A, Martinez JI, Chorpita BF, Weisz JR (2011). The youth self report: applicability and validity across younger and older youths. J Clin Child Adolesc Psychol.

[50] The Cochrane Collaboration. Review Manager (RevMan) [Computer program]; 2020.

[51] RStudio: Integrated Development Environment for R. Boston: RStudio Team; 2022.

[52] Petticrew M, Rehfuess E, Noyes J, Higgins JPT, Mayhew A, Pantoja T (2013). Synthesizing evidence on complex interventions: how meta-analytical, qualitative, and mixed-method approaches can contribute. J Clin Epidemiol.

[53] Morgan RL, Thayer KA, Santesso N, Holloway AC, Blain R, Eftim SE (2019). A risk of bias instrument for non-randomized studies of exposures: A users' guide to its application in the context of GRADE. Environ Int.

[54] Page MJ, Higgins JP, Sterne JA. Chapter 13: Assessing risk of bias due to missing results in a synthesis. In: Higgins JPT, Thomas J, Chandler J, Cumpston M, Li T, Page MJ, Welch VA, editor. Cochrane Handbook for Systematic Reviews of Interventions: version 6.3; 2022.

[55] Sterne JAC, Sutton AJ, Ioannidis JPA, Terrin N, Jones DR, Lau J (2011). Recommendations for examining and interpreting funnel plot asymmetry in meta-analyses of randomised controlled trials. BMJ.

[56] Egger M, Davey Smith G, Schneider M, Minder C (1997). Bias in meta-analysis detected by a simple, graphical test. BMJ.

[57] Schünemann HJ, Cuello C, Akl EA, Mustafa RA, Meerpohl JJ, Thayer K (2019). GRADE guidelines: 18. How ROBINS-I and other tools to assess risk of bias in nonrandomized studies should be used to rate the certainty of a body of evidence. J Clin Epidemiol.

[58] Schünemann HJ, Higgins JP, Vist GE, Glasziou P, Akl EA, Skoetz N, Guyatt GH. Chapter 14: Completing ‘Summary of findings’ tables and grading the certainty of the evidence. In: Higgins JPT, Thomas J, Chandler J, Cumpston M, Li T, Page MJ, Welch VA, editor. Cochrane Handbook for Systematic Reviews of Interventions: version 6.3; 2022.

[59] Mueller M, D'Addario M, Egger M, Cevallos M, Dekkers O, Mugglin C, Scott P (2018). Methods to systematically review and meta-analyse observational studies: a systematic scoping review of recommendations. BMC Med Res Methodol.

[60] Hilton Boon M, Thomson H, Shaw B, Akl EA, Lhachimi SK, López-Alcalde J (2021). Challenges in applying the GRADE approach in public health guidelines and systematic reviews: a concept article from the GRADE Public Health Group. J Clin Epidemiol.

[61] Harder T, Takla A, Eckmanns T, Ellis S, Forland F, James R (2017). PRECEPT: an evidence assessment framework for infectious disease epidemiology, prevention and control. Euro Surveill.

[62] Aromataris E, Stern C, Lockwood C, Barker TH, Klugar M, Jadotte Y (2022). JBI series paper 2: tailored evidence synthesis approaches are required to answer diverse questions: a pragmatic evidence synthesis toolkit from JBI. J Clin Epidemiol.

