# Supplementary material for: Anxiety among children and adolescents during the COVID-19 pandemic in Europe: a systematic review protocol
Source: Syst Rev. 2023 Apr 11;12:64. doi: 10.1186/s13643-023-02225-1 (PMC10088269; doi:10.1186/s13643-023-02225-1)
Supplement: Supplementary file 1 — Additional file 1: Appendix 1. Preferred Reporting Items for Systematic Review and Meta-analysis (PRISMA-P) 2015 checklist. Appendix 2. Peer Review of Electronic Search Strategies (PRESS) search submission form. Appendix 3. Peer Review of Electronic Search Strategies (PRESS) peer review assessment form. Appendix 4. Search strategy draft for PubMed. [file 13643_2023_2225_MOESM1_ESM.docx]

**Supplementary**

**Anxiety among children and adolescents during the COVID-19 pandemic in Europe: A systematic review protocol**

Helena Ludwig-Walz^a^, Indra DANNHEIM^b^, Lisa M. Pfadenhauer^c,d^, Jörg M. FEGERT^e^, Martin BUJARD^f,g^

^a^ Federal Institute for Population Research (BiB), Wiesbaden, Germany (HLW),
helena.ludwig-walz@bib.bund.de, ORCID: 0000-0003-1558-8241

^b^ Regional Innovative Centre of Health and Quality of Live Fulda (RIGL), Department of Nutrition, Food and Consumer Studies, Fulda University of Applied Sciences, Fulda, Germany (ID), indra.dannheim@oe.hs-fulda.de, ORCID: 0000-0002-4478-7808

^c^ Institute for Medical Information Processing, Biometry and Epidemiology - IBE, Chair of Public Health and Health Services Research, LMU Munich, Munich, Germany (LP), pfadenhauer@ibe.med.uni-muenchen.de, ORCID: 0000-0001-5038-8072

^d^ Pettenkofer School of Public Health, Munich, Germany (LMP), pfadenhauer@ibe.med.uni-muenchen.de, ORCID: 0000-0001-5038-8072

^e^ University Medical Center, Department for Child and Adolescent Psychiatry and Psychotherapy, Competence Domain Mental Health Prevention, Ulm, Germany (JMF), joerg.fegert@uniklinik-ulm.de, ORCID: 0000-0001-6070-4323

^f^ Federal Institute for Population Research (BiB), Wiesbaden, Germany (MB), martin.bujard@bib.bund.de, ORCID: 0000-0002-3603-4160

^g^ Institute for Medical Psychology, Medical Faculty, University Heidelberg, Heidelberg, Germany (MB), martin.bujard@bib.bund.de, ORCID: 0000-0002-3603-4160

Legend

[Appendix 1: Preferred Reporting Items for Systematic Review and Meta-analysis (PRISMA-P) 2015 checklist	2](#_Toc113967740)

[Appendix 2: Peer Review of Electronic Search Strategies (PRESS) search submission form	5](#_Toc113967741)

[Appendix 3: Peer Review of Electronic Search Strategies (PRESS) peer review assessment form	8](#_Toc113967742)

[Appendix 4: Search strategy draft for Pubmed	10](#_Toc113967743)

[References	11](#_Toc113967744)

# Appendix 1: Preferred Reporting Items for Systematic Review and Meta-analysis (PRISMA-P) 2015 checklist

[1]

| **Section and topic** | **Item No** | **Checklist item** | **Page in paper** |
| --- | --- | --- | --- |
| **ADMINISTRATIVE INFORMATION** | | |  |
| Title: |  |  |  |
| Identification | 1a | Identify the report as a protocol of a systematic review | 1 |
| Update | 1b | If the protocol is for an update of a previous systematic review, identify as such | not applicable |
| Registration | 2 | If registered, provide the name of the registry (such as PROSPERO) and registration number | 1, 2 |
| Authors: |  |  |  |
| Contact | 3a | Provide name, institutional affiliation, e-mail address of all protocol authors; provide physical mailing address of corresponding author | 1, 7 |
| Contributions | 3b | Describe contributions of protocol authors and identify the guarantor of the review | 7 |
| Amendments | 4 | If the protocol represents an amendment of a previously completed or published protocol, identify as such and list changes; otherwise, state plan for documenting important protocol amendments | not applicable |
| Support: |  |  |  |
| Sources | 5a | Indicate sources of financial or other support for the review | not applicable |
| Sponsor | 5b | Provide name for the review funder and/or sponsor | not applicable |
| Role of sponsor or funder | 5c | Describe roles of funder(s), sponsor(s), and/or institution(s), if any, in developing the protocol | not applicable |

| **INTRODUCTION** | | |  |
| --- | --- | --- | --- |
| Rationale | 6 | Describe the rationale for the review in the context of what is already known | 2 |
| Objectives | 7 | Provide an explicit statement of the question(s) the review will address with reference to participants, interventions, comparators, and outcomes (PICO) | Table 1 |
| **METHODS** | | |  |
| Eligibility criteria | 8 | Specify the study characteristics (such as PICO, study design, setting, time frame) and report characteristics (such as years considered, language, publication status) to be used as criteria for eligibility for the review | Table 2 |
| Information sources | 9 | Describe all intended information sources (such as electronic databases, contact with study authors, trial registers or other grey literature sources) with planned dates of coverage | 3 |
| Search strategy | 10 | Present draft of search strategy to be used for at least one electronic database, including planned limits, such that it could be repeated | Appendix 4 |
| Study records: |  |  |  |
| Data management | 11a | Describe the mechanism(s) that will be used to manage records and data throughout the review | 3 |
| Selection process | 11b | State the process that will be used for selecting studies (such as two independent reviewers) through each phase of the review (that is, screening, eligibility and inclusion in meta-analysis) | 3-4 |
| Data collection process | 11c | Describe planned method of extracting data from reports (such as piloting forms, done independently, in duplicate), any processes for obtaining and confirming data from investigators | 4 |
| Data items | 12 | List and define all variables for which data will be sought (such as PICO items, funding sources), any pre-planned data assumptions and simplifications | 4, Table 3 |
| Outcomes and prioritization | 13 | List and define all outcomes for which data will be sought, including prioritization of main and additional outcomes, with rationale | 4, Table 2, category “outcomes” |
| Risk of bias in individual studies | 14 | Describe anticipated methods for assessing risk of bias of individual studies, including whether this will be done at the outcome or study level, or both; state how this information will be used in data synthesis | 5 |
| Data synthesis | 15a | Describe criteria under which study data will be quantitatively synthesised | 5 |
|  | 15b | If data are appropriate for quantitative synthesis, describe planned summary measures, methods of handling data and methods of combining data from studies, including any planned exploration of consistency (such as I^2^, Kendall’s τ) | 5-6 |
|  | 15c | Describe any proposed additional analyses (such as sensitivity or subgroup analyses, meta-regression) | 5-6 |
|  | 15d | If quantitative synthesis is not appropriate, describe the type of summary planned | 6 |
| Meta-bias(es) | 16 | Specify any planned assessment of meta-bias(es) (such as publication bias across studies, selective reporting within studies) | 6 |
| Confidence in cumulative evidence | 17 | Describe how the strength of the body of evidence will be assessed (such as GRADE) | 6 |

# Appendix 2: Peer Review of Electronic Search Strategies (PRESS) search submission form

**SEARCH SUBMISSION: THIS SECTION TO BE FILLED IN BY THE SEARCHER**

| Searcher: | Email: |
| --- | --- |
| Date submitted: | Date requested by:  *[Maximum = 5 working days]* |

**Systematic Review Title:**

**This search strategy is…**

|  | My PRIMARY (core) database strategy — First time submitting a strategy for search question and database |
| --- | --- |
|  | My PRIMARY (core) strategy — Follow-up review NOT the first time submitting a strategy for search question and database. If this is a response to peer review, itemize the changes made to the review suggestions |
|  | SECONDARY search strategy— First time submitting a strategy for search question and database |
|  | SECONDARY search strategy — NOT the first time submitting a strategy for search question and database. If this is a response to peer review, itemize the changes made to the review suggestions |

**Database**

(i.e., MEDLINE, CINAHL…): *[mandatory]*

**Interface**

(i.e., Ovid, EBSCO…): *[mandatory]*

**Research Question**

(Describe the purpose of the search) *[mandatory]*

**PICO Format**

(Outline the PICOs for your question — i.e., Patient, Intervention, Comparison, Outcome, and Study Design — as applicable)

| **P** |  |
| --- | --- |
| **I** |  |
| **C** |  |
| **O** |  |
| **S** |  |

**Inclusion Criteria**

(List criteria such as age groups, study designs, etc., to be included) *[optional]*

**Exclusion Criteria**

(List criteria such as study designs, date limits, etc., to be excluded) *[optional]*

**Was a search filter applied?**

Yes □	No □

**If YES, which one(s) (e.g., Cochrane RCT filter, PubMed Clinical Queries filter)? Provide the source if this is a published filter.** *[mandatory if YES to previous question* — *textbox]*

Other notes or comments you feel would be useful for the peer reviewer? ***[optional]***

Please copy and paste your search strategy here, exactly as run, including the number of hits per line. ***[mandatory]***

(Add more space, as necessary.)

# Appendix 3: Peer Review of Electronic Search Strategies (PRESS) peer review assessment form

**PEER REVIEW ASSESSMENT: THIS SECTION TO BE FILLED IN BY THE REVIEWER**

| **Reviewer:** | **Email:** | | **Date completed:** |
| --- | --- | --- | --- |
| **1. TRANSLATION** | | | |
|  | A. No revisions | **□** |  |
|  | B. Revision(s) suggested | **□** |  |
|  | C. Revision(s) required | **□** |  |

If “B” or “C,” please provide an explanation or example:

| **2. BOOLEAN AND PROXIMITY OPERATORS** | | | |
| --- | --- | --- | --- |
|  | A. No revisions | **□** |  |
|  | B. Revision(s) suggested | **□** |  |
|  | C. Revision(s) required | **□** |  |

If “B” or “C,” please provide an explanation or example:

| **3. SUBJECT HEADINGS** | | | |
| --- | --- | --- | --- |
|  | A. No revisions | **□** |  |
|  | B. Revision(s) suggested | **□** |  |
|  | C. Revision(s) required | **□** |  |

If “B” or “C,” please provide an explanation or example:

| **4. TEXT WORD SEARCHING** | | | |
| --- | --- | --- | --- |
|  | A. No revisions | **□** |  |
|  | B. Revision(s) suggested | **□** |  |
|  | C. Revision(s) required | **□** |  |

If “B” or “C,” please provide an explanation or example:

| **5. SPELLING, SYNTAX, AND LINE NUMBERS** | | | |
| --- | --- | --- | --- |
|  | A. No revisions | **□** |  |
|  | B. Revision(s) suggested | **□** |  |
|  | C. Revision(s) required | **□** |  |

If “B” or “C,” please provide an explanation or example:

| **6. LIMITS AND FILTERS** | | | |
| --- | --- | --- | --- |
|  | A. No revisions | **□** |  |
|  | B. Revision(s) suggested | **□** |  |
|  | C. Revision(s) required | **□** |  |

If “B” or “C,” please provide an explanation or example:

| **7. OVERALL EVALUATION (Note: If one or more “revision required” is noted above, the**  **response below must be “revisions required”.)** | | | |
| --- | --- | --- | --- |
|  | A. No revisions | **□** |  |
|  | B. Revision(s) suggested | **□** |  |
|  | C. Revision(s) required | **□** |  |

Additional comments:

# Appendix 4: Search strategy draft for Pubmed

| **Category** | **Search terms** |
| --- | --- |
| **P**opulation | Infan* OR toddler* OR minors OR minors* OR boy OR boys OR boyfriend OR boyhood OR girl* OR kid OR kids OR child OR child* OR children* OR schoolchild* OR schoolchild OR school child[tiab] OR school child*[tiab] OR adolescen* OR juvenil* OR youth* OR teen* OR under*age* OR pubescen* OR pediatrics[mh] OR pediatric* OR paediatric* OR peadiatric* OR school*[tiab] OR kindergarten*[tiab] OR kindergarden*[tiab] |
| **E**xposure | ("Covid-19"[Mesh] OR covid*[tiab] OR "SARS-CoV-2"[Mesh] OR “sars-2”[tiab] OR “sars2”[tiab] OR “sars-cov-19”[tiab] OR “sars-cov19”[tiab] OR “sarscov-19”[tiab] OR “sarscov19”[tiab] OR "SARS-CoV-2"[tiab] OR “sarscov-2”[tiab] OR “sars-cov2”[tiab] OR “sarscov2”[tiab] OR "SARS CoV 2"[tiab] OR "Severe Acute Respiratory Syndrome" [tiab] OR "Severe Acute Respiratory disease"[tiab] OR "coronavirus"[MeSH] OR Coronavirus*[tiab] OR corona-virus*[tiab] OR “corona virus*”[tiab] OR ncov*[tiab] OR n-cov*[tiab] OR “n cov”[tiab] OR novelcov*[tiab] OR novel-cov*[tiab] OR “novel cov*”[tiab]) AND (2019/11/01[PDAT] : 3000/12/31[PDAT]) |
| **C**omparison | Not applicable |
| **O**utcome | "Anxiety" [Mesh] OR “Anxiety Disorders”[Mesh] OR anxi*[tiab] |
| **S**pecies | FinalResult NOT (animals[MeSH Terms] NOT humans[MeSH Terms]) |

References

1.	Shamseer L, Moher D, Clarke M, Ghersi D, Liberati A et al. (2015) Preferred reporting items for systematic review and meta-analysis protocols (PRISMA-P) 2015: elaboration and explanation. BMJ (Clinical research ed.) 350: g7647.
